# Exploring disconnected discourses about Patient and Public Involvement and Volunteer Involvement in English health and social care

**DOI:** 10.1111/hex.13162

**Published:** 2020-12-01

**Authors:** Jurgen Grotz, Linda Birt, Heather Edwards, Michael Locke, Fiona Poland

**Affiliations:** ^1^ Institute for Volunteering Research University of East Anglia Norwich UK; ^2^ School of Health Sciences University of East Anglia Norwich UK; ^3^ Come Singing Norwich UK; ^4^ Riding for the Disabled Association, Greater London Region London UK; ^5^ School of Health Sciences University of East Anglia Norwich UK

**Keywords:** volunteering, health and social care, NHS, patient and public involvement, voluntary action, social theory, social policy, policy discourse, discourse analysis

## Abstract

**Background:**

Patient and public involvement (PPI) in health and social care policy, service decision‐making and research are presented as good practice in England. Yet the explicit rationale for PPI and how it is positioned within the literature, policy and practice remain confused, in particular, in relation to Volunteer Involvement (VI). In health and social care, PPI and VI are managed and valued as conceptually distinct, yet the discourses in their policy and practice documents treat them as closely related in fundamental ways.

**Objective:**

Compare and critically evaluate discourses framing PPI and VI within English health and social care.

**Design:**

A critical discourse approach was used to explore the accounts of PPI and VI in policy. These accounts were then compared and contrasted with personal accounts of volunteering in health and social care settings.

**Results:**

Twenty documents from key national health and social care bodies were discursively examined in terms of their framing PPI and VI. A narrative disconnect between the two was repeatedly confirmed. This finding contrasted with an analysis of personal accounts of VI which displayed VI as a form of PPI.

**Conclusion:**

There is a disconnect between language, narratives and practice in PPI and in VI which may have direct consequences for policy and practice. Recognising and managing it can offer innovative ways of enabling volunteers to be involved across health and social care settings, ensuring the experiential value added by volunteers’ service contributions, to be recognised so that their democratic participation may be seen to shape services.

## 1 INTRODUCTION

There are an estimated three million volunteers annually in health and social care in England.^1^ A growing number of these are volunteer patient and public involvement (PPI) members. Since the millennium, there has been an increase in numbers of PPI members in research projects. In some policies and guideline documents, volunteers and PPI members are discussed in terms of their roles and contributions being clearly distinct and separate. However, practices in PPI, for example regarding reciprocal relationships between researchers and PPI members, presented as novel findings in PPI research,^2^ have long been recognised as basic to good practice in managing and empowering volunteers.^3^ This indicates a potential disconnect between the discourses of PPI and VI in health and social care. If this is so, those involved in organising and supporting PPI and VI may fail to appropriately draw on sources of practice‐based knowledge. This paper critically examines connection and disconnection in policy discourses relating to both PPI and VI. To do this it draws on Gee’s^4^ approach to discourse analysis to link language used in characterising PPI and VI within exemplar policy documents, stories, actions and discourse communities, to social practices.

The idea for this review was developed through discussion with lay advisory members of the Institute for Volunteering Research during informal review of National Health Service [NHS] policies and workshops undertaken to explore the place of VI in health and social care. Of the NHS documents informally reviewed, about half recognised people involved in designated PPI activities, such as GP patient participation group members, as volunteers ^5^. However, it was striking that the other half of these documents presented as principally concerned with PPI, such as NHS England *Patient and Public Participation Policy*, made no mention at all of volunteering.^6^ This preliminary work suggested a potential discursive disconnect affecting the conceptualisation of practice might need to be further evidenced.

This paper reports the exploratory approach we undertook to explore the tentative proposition that there was disconnect between the discourse of policy and the pragmatics of practice between both PPI and VI. To situate our method, results and discussion we first account for our working definitions of PPI and VI.

We acknowledge PPI is a contentious term but here we use the term to reflect the activity of patients and public who move from being unquestioning recipients of services to be involved citizens in the planning and delivery of health and social care for all.^7^ PPI in health research and service decision‐making is commonly described as ‘imperative’ for improving patient experiences.^8^ However, following decades of implementing PPI, a marked lack of agreement or even shared understanding of what it is and should be persists among those interested in PPI or even those involved in it.^9^, ^11^ A repeated criticism of PPI is its being conducted as little more than a ‘tick box exercise’.^10^ While it is seen to be embedded in practice, in varying ways and degrees, this is rarely comprehensive, and so presents as having negligible impact.^9^, ^11^ Locock et al^13^ ^(p. 836)^ also point to research into PPI as being conceptually and theoretically poor, reducing our knowledge to its “mechanisms” and “contextual elements.” This suggests the need for further discursive critique of PPI in health and social care policy documents.

In this paper we define VI as involving a commitment of time and energy which can take many forms but is undertaken by choice and without concern for financial gain, conferring wider benefit.^14^ VI in health and social care has grown alongside the activity of involving the public in service decision‐making. Both PPI and VI are broadly conceptualised as ‘service’^15^ in which the volunteers ‘give freely’ of their time.^1^, ^16^ PPI and VI in health and social care settings are often described as instrumental and such a discourse fails to encompass the affective values conveyed through volunteering, which include the intrinsic democratic nature of volunteering.^17^ Rochester et al^18^ have described and criticised the focus on service volunteering as the ‘dominant paradigm’. This dominant paradigm frames volunteering as playing a passive and subordinate role in services while failing to recognise that those who volunteer regularly make and have to make active autonomous decisions, and also influence service providers’ decision‐making. There is a need to more robustly explore whether volunteers influence service provision in health and social care.

The current significant policy drives to swiftly and hugely increase PPI and VI in health and social care yet practice frequently takes place in a context in which PPI is still often seen as a tokenistic exercise demanded by policy, contrasted with volunteering as reducible to unquestioning service provision, and with each contribution positioned as distinct from the other.

To explore any such conceptual deficit and to develop means to address it if so, we aim to critically review recent relevant policy documents to consider whether and how we might better conceptualise both PPI and VI if we acknowledge the connections between both activities which may offer a more appropriate novel theoretical approach. Using a critical discursive approach, the paper starts by separately examining policy discourses relating to PPI and to VI. We then present three case studies of personal experiences of volunteering involvement, critically appraising these to see if volunteering can be framed as actively shaping health and social care practices, which would be enacting features of PPI.

## 2 METHOD

We adopted a deductive exploratory approach seeking to confirm or contradict the proposition, developed from our early discussions and informal review that there was a disconnect between PPI and VI discourses in published literature and policy relating to health and social care in England. We drew on principles of critical discursive analysis to examine in detail whether and how distinct meanings may have been allocated to the two activities of PPI and VI as reported within policy documents and through personal narratives. Discourses are the texts and talk which support shaped understandings of meaning.^19^ A discursive analysis approach enables the study and critique of how meaning‐making is socially constructed through the language used by discourse communities to help organise and present their changing social activities within interactions and over time.^20^ Health and social care encompass the practices of many different groups of service users and providers interactively engaging with diverse needs and expectations of what service provision may be appropriate. In this context, it is therefore vital to understand how meanings for practice may be deployed, to realise for what purposes, and whether meanings are being culturally shared across PPI and VI activities and policies or may underpin disconnections between them.

To identify and then build understandings of ways in which PPI may be currently presented as conceptualised within health and social care organisation policy rhetoric, we searched policy documents from key organisations involved in health and social care services such as NHS England, Health Research Authority, National Institute for Health Research (NIHR) and Healthwatch; retrieving review policy documents published between 2012 and 2019. This time period corresponded to the English Health and Social Care Act 2012 which created a ‘duty for public involvement’ until the most recent possible completion date, December 2019. Polices were selected if they had national influence and referred to public involvement and/or volunteering in health and social care.

To similarly explore current conceptualisations advanced in the discourse on VI, we examined definitions commonly used in standard volunteering and volunteer texts, to add to definitions including the purpose of volunteering, current policy changes and volunteer involvement practices in health and social care. Two management textbooks^3^, ^18^ were selected as they are recommended reading in training for volunteer mangers and complement NHS guidance. The selected policy documents offer a subject‐specific view from the Kings Fund, a recognised reviewer of health and social care in England^1^, ^16^ and three highly cited journal articles which define VI.^21^, ^22^, ^23^

We purposively select diverse exemplar case studies from a pool of accounts collected to support participant discussions for the Institute for Volunteering Research. Seven collaborative events were held between October 2018 and July 2019 designed to explore the positioning of VI within the context of PPI. These cases were purposefully selected as directly relating to VI as identified by NHS England examples of volunteer's roles ‘Entertainment/music/arts and crafts/ activities’, ‘Support with long‐term conditions’, ‘GP patient participation group’^5^ ^(p. 5)^ and as relating to three very different service settings: in a hospital, in primary care, and a social care activity. These provided critical comparative case materials, on which Grotz and Poland carried out the initial formal discourse analysis. All authors then deliberated the framing of VI and PPI terms and purposes in each account and in the volunteering settings to which they related.

This research activity was not submitted for formal review by an ethics committee. However, the authorial team considered ethical issues throughout. Ethical issues most closely considered included particularly those of consent relating to access to and use of personal information and differences in power to control those uses within this research activity. The policy documentary analysis analysed only publicly available documents, which did not require formal consent to read and comment on. The case accounts were specifically offered for analysis here by the co‐authors who had generated them and who would themselves be co‐framing and providing final comments on their use.

## 3 RESULTS

We present the results of policy discourse analysis under two headings: policy discourses on PPI, policy discourses on VI. We then present case studies on VI in health and social care considering how they may additionally foreground PPI elements or not, to help identify and characterise how any narrative disconnect between VI and PPI is constructed and to contextualise reasons for this.

### 3.1 Exploring the policy discourses of patient and public involvement (PPI) in health and social care

Twenty documents, relevant to policy in England, were identified and selected, as presenting definitions, statements of purpose, and practice statements for PPI as a form of public involvement, and then considered in terms of how they did or did not relate to volunteering purposes. Policy documents were drawn from UK health departments, the Health Research Authority, NHS England, NIHR and INVOLVE (see Table 1).

**Table 1:** Summary of documents reviewed and whether including explicit reference to VI and PPI

Ref	Date	Document	PPI=VI	PPI≠Vl
^24^	2012	Health and Social Care Act (2012), chapter 7		X
^25^	2012	INVOLVE (2012) Briefing notes for researchers: public involvement in NHS, public health and social care research.		X
^26^	2014	NIHR (no date) *Patient and public involvement in health and social care research: A handbook for researchers*, NIHR, no place		X
^6^	2017	NHS England (2017) Patient and public participation policy. NHS England: Redditch		X
^27^	2017	NHS England (no date, a) Involving people in their own health and care: Statutory guidance for clinical commissioning groups and NHS England. NHS England, no place		X
^28^	2017	NHS England (no date, b) Patient and public participation in commissioning health and care: Statutory guidance for clinical commissioning groups and NHS England. NHS England, no place		X
^29^	2018	NIHR (2018) National Standards for Public Involvement in Research. NIHR: London		X
^30^	2015	NHS England (2015) The NHS Constitution: the NHS belongs to us all, Department of Health, no place	X	
^31^	2017	NHS England (2017) Working with our Patient and Public Voice (PPV) Partners – Reimbursing expenses and paying involvement payments (v2). NHS England: Redditch	X	
^5^	2017	NHS England (2017) Recruiting and managing volunteers in NHS providers, a practical guide. NHS England, no place	X	
^32^	2017	Health Research Authority (2017) UK policy framework for health and social care research v3.3 07/11/17	X	
^15^	2017	Health Research Authority (2017) Health Research Authority Annual Report and Accounts For the Year to 31 March 2017	X	
^33^	2018	Health Research Authority (2018) Health Research Authority Annual Report and Accounts For the Year to 31 March 2018	X	
^34^	2018	Health Research Authority (2018) Governance arrangements for research ethics committees: 2018 edition	X	
^35^	2017	Healthwatch (2017) Healthwatch England Annual Report 2016‐17. Healthwatch England, no place	X	
^36^	2018	Healthwatch (2018) Healthwatch England Annual Report 2017‐18. Healthwatch England, no place	X	
^37^	2019	Department of Health and Social Care and Public Health England, website, 2019 Guidance Handbook to the NHS Constitution for England Updated 28 October 2019. Department of Health and Social Care and Public Health England	X	
^38^	2019	NHS Website (no date) *About the NHS ‐ getting involved in the NHS*	X	
^39^	2019	Health Research Authority (2019) Health Research Authority Annual Report and Accounts 2018/19.	X	
^40^	2019	NHS long‐term plan	X	

This set of documents is first located within their historical context, then their use of PPI (and VI) terminology across the documents and within the organisations is considered, and finally any commonly stated purposes for using terminology in the ways they do are identified.

A persisting policy trend to democratise health and social care, since the UK Prime Minister Margaret Thatcher’s *Patient Charters* in the 1980s, can be seen as intended to contribute to a wider discursive challenge to the National Health Service monopoly (NHS).^41^ This was later restated in Prime Minister Tony Blair’s approach to ‘Shifting the Balance of Power within the NHS’^42^ which set out reforms and intended NHS performance improvements. An intention to democratise health and social care was more recently articulated in the Health and Social Care Act (2012) by the UK’s Conservative‐Liberal Democrat Coalition government, then updated in 2016 under the next (Conservative‐only) Government.

The Health and Social Care Act (2012) placed legally binding, wide‐ranging duties on NHS England and on Clinical Commissioning Groups to ensure individuals were involved in decision‐making by being consulted and given information or in other ways.^24^ This statutory requirement to involve has shaped the formal public commitments of health services providers, including the four nation‐devolved UK Health Departments, to create shared explicit policies, structures and practices to support patients, service users and the public ‘to get involved in its design, management, conduct and dissemination, and [be] confident about doing so’.^32^ ^(p. 4)^

The statutory requirement for PPI within the Health and Social Care Act (2012) may be linked not just to wider policy purposes to democratise but also to respond to various health scandals revealing organisational mistakes and negligence.^43^ While there are criticisms of how democratic and supportive of involvement the legislation has been in practice,^44^ public involvement is now a clearly stated policy enacted in many health and social care provider organisations.

While this part of the review indicates a narrative acceptance of PPI within the wider policy context, some criticisms of PPI are also advanced.

These documents provided little firm agreement about what terms are best to use to describe practices of involving people in health and care services. The NIHR handbook for researchers in health and social care research notes that PPI is also known as ‘service user or lay involvement’.^26^ NHS England guidance on working with Patient and Public Voice Partners suggests that partners may also be referred to as people participating in ‘service user involvement’, as ‘lay representatives’, ‘lay voices’, ‘public voice representatives’ or ‘patient and public involvement (PPI) representatives’.^6^, ^31^ Diverse meanings of PPI could be seen to generate tensions between statutory health‐related bodies. INVOLVE, a national advisory group until recently part‐funded by NIHR, markedly contrasted with other stated NHS England policies, in explicitly excluding from such involvement, activities referred to as ‘engagement’ and ‘participation’. Perhaps, this position is now changing as a new NIHR centre for patient and public involvement, engagement, participation and research dissemination has been established in April 2020.

Applying the INVOLVE position to general guidance on involvement would mean excluding activities aiming to raise awareness of research, sharing knowledge, engaging in dialogue with the public or being a study participant‐subject.^19^ INVOLVE’s restriction of the term ‘participation’ to people recruited as study subjects, and still avoided in the UK’s recent National Standards for Public Involvement,^29^ conflicted with UK Health Research Authority^15^ use of terms and also with other NHS England guidance such as on commissioning. In the latter case, the term ‘involvement’ is used interchangeably with rather than contrastively with ‘engagement’, ‘participation’, ‘consultation’ and ‘patient or public voice’.^28^ ^(p. 9)^

Similarly, when referring to the ‘public’, a frequently used definition is ‘patients, potential patients, carers and people who use health and social care services as well as people from organisations that represent people who use services’.^25^ ^(p. 6)^ Recognising here that everyone is a potential patient seems to then mainly differentiate perspectives to reflect the distinctive standpoints of those with or without a professional role in health and social care.

On the second challenge for coherently critiquing PPI purposes, two purposes are commonly presented as informing public involvement in practice in NHS England: promoting inclusivity and improving decision‐making. For example, in the participation policy of NHS England,^6^ four of its ten declared principles focus on the need for inclusion and better decision‐making, while the remaining six principles define how this may be achieved. In the NIHR national five standards for public involvement, the first concerns inclusivity and the remaining four concern working in ‘mutually respectful and productive partnerships’ to ‘promote and protect the public interest’ in decision‐making.^29^ ^(p. 5)^

In summary, while we can ascertain the policy origins of PPI and some agreement on the stated purposes which can match that origin, there is little clear statement throughout the policy documents reviewed, as to whose purposes these would advance and how to achieve them.

### 3.2 Exploring the policy discourses of volunteer involvement in health and social care

As in the previous section, the analysis here is first set into the historical context of key changes since the introduction of the NHS, again identifying key interconnecting features and specific problem areas these raise. We consider their use of terminology the in‐policy discourse and again identify any commonly stated purposes.

Historically, Beveridge’s seminal 1948 work on voluntary action defined it relating to activities ‘not under the direction of any authority wielding the power of the State’,^45^ ^(p. 8)^ and so unlikely to take place in the public sector. Since then, NHS England has changed the discourse dramatically, recently declaring that a ‘large number of voluntary, community or private sector organisations work in partnership with the NHS and involve volunteers in NHS settings’.^5^ ^(p. 5)^ Services actually provided by volunteers are identifiable in accounts of service practice across hospitals, primary care and general practice and in community settings. Activities are often described as ‘patient‐centred’, while ranging widely in type to include befriending, peer support for breast‐feeding and helping patients mobilise. There are also many examples of volunteering which do not involve direct patient contact, again ranging widely from fund‐raising and delivering supplies to monitoring security.^5^, ^16^

As with PPI, service providers have expressed their commitment to greatly increase the volume of volunteering activities, indeed to ‘double the number of NHS volunteers over the next three years’.^40^ ^(p. 90)^ Modest estimates suggested three million people were already volunteering in health and social care in 2013.^1^ ^(p. 5)^ The drive to increase VI, specifically the numbers of volunteers in new settings such as in the NHS Responder scheme, has most recently been accelerated as sudden health and social care demands have been stimulated by the expectations and consequences of the coronavirus pandemic.

The NHS provides substantial guidance on how to frame VI practice in its guide to ‘Recruiting and managing volunteers in NHS providers’.^5^ This guide lays out specific principles of best practice for involvement, to explicitly include ‘GP patient participation group’, ‘Expert patients’, and ‘Governors and trustees’.^5^ ^(p. 5)^ In line with standard practice,^3^ the NHS England guidance articulates core volunteer management principles around recruitment and retention, marked by a commitment to forms of VI which recognises the welfare of the volunteers and their contribution. However, as in characterising PPI, unresolved disagreements remain between core definitions and concepts of volunteering articulated.^18^

The commonly presented purposes centre on the shared aim of facilitating collaborative connections between people and organisations. However, these fall short of other kinds of contractual arrangements to enable mutually beneficial inclusive activities which might offer service improvements in health and social care.^40^ ^(p. 90)^ The policy discourses in the reviewed documents do not reflect the range of purposes of VI recognised in the wider literature.^21^, ^22^, ^23^, ^46^, ^47^

In summary, we can discern a significant policy discourse shift over the last decades to include VI as a means to deliver services in health and social care. Nonetheless such policy discourses are not mutually consistent in recognising that accounts of VI and PPI practice do implicitly, not explicitly, reflect issues and concerns in VI discourses more widely. We now apply these discursive insights to analyse three critical cases provided by personal reflective accounts of volunteering in health and social care, (Locke and Edwards, who did not contribute to the specific analysis of the accounts they produced), to examine whether and how they articulate any wider purposeful contribution of volunteering than service delivery.

### 3.3 Personal reflective accounts of volunteering in health and social care

The commonly stated purposes of PPI and VI identified in the policy discourse analysis were as follows: inclusivity, improving decision‐making and shaping service improvements in health and social care. The following three case studies were selected as they directly related to diverse activities identified by NHS England guidance^5^ ^(p. 5)^ as forms of volunteering in health and social care, to enable further considerations of how volunteers themselves in these areas deploy discourses within their different areas of volunteering but which may articulate commonly stated purposes of PPI and VI identified here in policy discourses.

The first case study is provided by a person whose personal love of and skill in music has led them to offer to develop this as a volunteering activity in a local hospital. Using and making music is a recognised volunteer activity within the NHS ‘Entertainment/music/arts and crafts/ activities’.^5^ ^(p. 5)^ This case study highlights how the volunteer’s account presents the direct impact of volunteering on service improvement for a patient with aphasia.

#### 3.3.1 Case study 1 Providing music in hospital: shaping service improvements

I volunteer at my local general hospital; I play music on a keyboard set up in the main corridor of orthopaedic, stroke/neurological and acute medical wards. The repertoire generally includes light classical, folk, traditional, songs from the musicals and jazz standards to provide pleasant familiar ambient music. I respond to individual patient or staff engagement as it happens.

One such instance was with a younger patient who had been on the neurological ward for some weeks with aphasia. She was alternately restless and sleepy and had shown no interest in music. A session was winding down with some childhood favourites when ‐ seemingly by chance ‐ she began joining in with 'My Bonny lies over the ocean' and found that she could enunciate some of the words. Surprise and excitement were obvious: she began tugging at the sleeve of the carer, gesturing to her own lips, and finally with huge effort shouted loudly 'It's…tremendous!' Ward staff were clearly delighted and taken aback, and a junior doctor commented that he had 'never seen her so well.'

In between my fortnightly visits the ward sister rang to say that she was keen to build on the incident. The patient herself later recognised me (even without keyboard) as 'Mrs. Songs' and again managed to find words to call out a greeting, adding that 'It was great ‐ we loved it.' In a subsequent music session, she found that she could read aloud fluently from a printed song sheet, without singing.

Staff were intrigued, surprised and encouraged by these developments as the patient still had no fixed diagnosis. On her discharge for further assessment it was arranged that I should continue to visit with music.

While they did not present their activity as PPI, this case study depicts the impact of H’s VI activity in service improvement, by impacting on how health professionals perceived the patient and the nature of the care they were provided. In the general routine of care, the patient was noted to have little interest in music activity, but the trigger of a familiar song, provided through VI, was shown as sparking her verbal communication. The activity is presented as giving the volunteer a means to impact directly not only on their relationship with patients but also on reshaping their care provision, a reshaping which the hospital staff are described as supporting and further sustaining patient assessment. This example illustrates ways in which the volunteer involvement is reframed as going beyond simply providing a service. This sharply contrasts the agentic volunteering contribution in practice against the dominant paradigm of simply providing services set elsewhere.

In this second case study, the volunteer activity takes place in social care in the context of providing riding for disabled people. This type of activity is embedded within the health service category of ‘Support with long‐term conditions’.^5^ ^(p. 5)^ The charity Riding for the Disabled Association (RDA) is a federation of 500 independent groups ‘committed to providing life‐changing experiences for disabled children and adults’ with equestrian activities.

This case study highlights how volunteering can open up new opportunities for accessing activity and experiences not only to service users but also to volunteers, and so addressing multiple types of inclusion.

#### 3.3.2 Case study 2 Enabling riding for disabled people: enabling inclusion

Initially my volunteering in RDA was a basic service role: sweeping the yard; poo‐picking; serving drinks at fund‐raising events. I did what I was asked because I saw the value of the activities and I liked the people.

I had no role in service‐design or decision‐making. My observation was that those functions belonged with people who held expert knowledge in horsemanship, who could apply that knowledge to children and adults with distinct disablements and capabilities. I was working within top‐down structures within the riding centre and across the horse world.

Two things changed for me when I was appointed to chair the Greater London Region of RDA, which cast me into an expert role. Although my expert knowledge was in voluntary organisations rather than therapeutic work, in the role I learned to join in expert decision‐making. Concurrently, my involvement in the riding centre widened to include direct support of children while riding. I was ‘side‐walking’, walking beside a disabled child on a horse, particularly to guard against an accident. Here I developed skills in communicating with the child, whether to help them focus on the lesson or to build conversation as they relaxed through riding. With one boy I explored how to identify colours of cones and then, seeing that he could find decision‐making difficult, I helped him make decisions about which colour to ride to. Thus, while clearly an assistant, I took more of a role in decision‐making. I devised an individual lesson plan for him within the generic group lesson plan developed by the coach. This one‐to‐one experience informed my strategic view of RDA, providing me with evidence that lessons were consistent with RDA aims for enhancing the quality of coaching across the organisation.

I have found the roles of service‐volunteering and of expert decision‐making to be interwoven in complex ways, rather than existing in parallel or hierarchical dimensions.

In this example, VI is again not explicitly presented as PPI, but can be clearly related to the identified aims of PPI to offer inclusive (non‐hierarchical) and better decision‐making. The volunteer’s account also identifies the different types of public involvement they recognised themselves as accessing and then enacting in their volunteer role, as a service provider, then a decision taker, then a decision enabler. All these roles foreground the actively involving service users’ experience, another key proposed feature of PPI not VI. However, as an explicitly VI activity, much PPI discourse would not recognise and reinforce these convergent links and so not provide a discursive basis to affirm the resonance of such activities with PPI.

The third example is drawn from a type of activity in primary care also identified and proposed by NHS England: ‘GP patient participation group’.^5^ ^(p. 5)^ Since 2015, it has been mandatory that each GP practice has a Patient Participation Group (PPG) to provide critical commentary on practice policies and issues. This is defined and advertised as a volunteer position, and also identifies participation in organisation decision‐making from the start. It is a recognised VI role which specifies setting out to explicitly address a PPI purpose.

#### 3.3.3 Case study 3 Adding the patient viewpoint: improving decision‐making

I was invited to join my Doctor’s PPG group by a friend who was already a group member. The commitment was not too heavy with regular quarterly meetings and I appreciated the efficiency of my local GP practice so I agreed. My role in the group is to monitor and advise. At each meeting we are given an update on practice‐related issues such as staff appointments, missed appointment, government monitoring. As a volunteer I provide a patient perspective and this can be most helpful when discussing patient comments, how to spend money donated by patients and how to deal with missed appointments. For example, the GPs wanted to take action with patients who repeatedly fail to attend. Several local practices had the policy that after three‐missed appointment patients were removed from the GP list. This policy did not fit the ethos of our practice so we agreed repeat offenders would need to phone on the day and be nurse triaged. In this role I have had the chance to attend CCG events and have increased my understanding of health delivery which support me in my other volunteer role as a local councillor.

In this example, the volunteer explicitly links VI and PPI, presented as VI, which offers the volunteer a role to help shape health and social care services, presented as leading to the formally stated effect of better, and more inclusive, decision‐making.

These three examples demonstrate how highly diverse accounts of activities of VI in health and social care nonetheless share ways of presenting volunteers or present themselves, to include experiencing themselves playing an active part in shaping and monitoring services through their volunteering. This means they are also presented as playing an important and grounded part in democratising those services from below.

These three critical examples show diverse activities as depicting both PPI and VI purposes. These accounts counteract those discourses, which treat PPI and VI as distinct, seen in discursive review of the formal policy documents supporting our proposition that there is a disconnect between policy and practice in relation to PPI and VI.

We now move to critically discuss such disjunctures within the PPI and VI discourses and between the dominant discursive paradigm of PPI and VI as service, and distinct from democratic participation processes, linking PPI and VI. We then reflect on the related practice implications for both.

## 4 DISCUSSION

PPI and VI are presented by policy makers, commissioners, service providers and academics as activities distinct from each other with different rules and customs. However, the inconsistencies and conceptual deficits of definition and resonance within and across organisations are starkly exposed by both types of data and analysis presented here.^5^, ^6^, ^20^ Comparatively examining the discursive distinctions drawn between PPI and VI in the discourses examined, to reflect on how groups and organisations do and can develop policy and practice, is severely constrained by how inconsistently and interchangeably terminology is used in documentation and by policy makers which make explicit affirming shared purposes for PPI and VI practice, while articulating sharply differing concepts to discursively present each.

### 4.1 PPI is a type of VI

Our first finding is that, when identifying how the VI discourse is applied to PPI, PPI can be framed as a type of VI. PPI is described in terms which clearly fall within the dominant definitions and taxonomy of volunteering, in particular, as activities which are non‐coerced, unpaid or not undertaken mainly for financial reward, outside one’s own family and conferring wider benefit.^18^ ^,^ ^46^ Furthermore, the findings in 13 out of 20 documents, from our review of PPI‐related policies, evidenced clear discursive recognition across NHS England, HRA and Healthwatch of PPI activities presented as VI. However, seven out of 20 reviewed policy documents do not show any such discursive recognition including some from NHS England and all reviewed documents published by the NIHR. This might reflect an NIHR claim that research subjects should be clearly demarcated from lay people and service users involved in governance and to allocate a particular status to them. However, any consultation even within key national policy and services providers does not seem to have resulted in any such shared clarity on terminology or status.

Alternatively, if we position PPI explicitly as a commonly recognised feature of VI within health and social care, as found in the case study accounts, this delineates a wider arena of theory which can explore all such activities. This would widen the conceptual context to extend the current narrow view of PPI, as a distinct but under‐conceptualised activity, to access a clearer and more robustly founded taxonomy and theory. This, in turn, would more sharply demarcate the claims of PPI while locating its status within organisations and the wider world of volunteering. Such a discursive resolution is apparently sought and certainly needed, given the constantly expressed regrets about lack of agreement or shared understanding among PPI interest groups, about what PPI is and should be.^9^, ^10^, ^11^

### 4.2 Currently not‐included forms of VI can be PPI

Our second finding is that many VI activities in health and social care, currently not included, can be conceptualised and framed as PPI. Our review found not only that the language of PPI policy resonates with VI as illustrated above but that the VI discourse around a ‘civil society paradigm’^46^ also aligns with the PPI discourses. One of NHS England’s documents, on patient and public involvement, categorises the involvement of patients and the public in terms of four types of volunteering roles:

responding to or commenting on open access engagement opportunities

attending workshops / events / focus groups on a ‘one off’ basis

becoming a member of regular working group

taking on a senior Expert Advisor role that demonstrate strategic and accountable leadership and decision making activity.^31^ ^(p. 8)^

These roles are also reflected in the lived experience narratives of the volunteer case studies which in places resemble aspects commonly assumed to apply only to PPI roles. In Case Study 2, for example, the volunteer takes on an expert role which entails accountable leadership and decision‐making. In Case Study 3, the volunteer is a member of a regular working group explicitly involved in decision‐making about primary healthcare services, rather than directly providing those services.

Our analysis of the VI policy discourse and of our case studies challenges the dominant ‘service delivery’ paradigm of VI in health and social care, to show that VI can be widely recognised by staff and other participant in those settings as much more wide ranging than just delivering a service, and as already distinctively contributing to shaping and monitoring health and social care.

As pointed out above, much of current PPI discourse is specifically rooted in the policy trend to democratise health and social care, which is also exemplified by the opening statement of the NHS constitution: ‘The NHS belongs to us all’.^30^ ^(p. 1)^ The terms of the NHS constitution may present their designation, to be interpreted to mean the public will be democratically involved, not just in delivering, therefore, but also in shaping and monitoring those services. The exemplary cases of accounts of volunteering in health and social care make explicit that all VI in health and social care is provided by actual or potential patients. Where their involvement as volunteers can be seen, as here, to also shape and monitor those services, it may therefore also be viewed as another part of the intensifying policy and social drive to ensure democratic participation. We can apply these insights to address the full spectrum of volunteering, to even include the concepts of volunteering, for mutual aid, for self‐help and for leisure.^48^, ^49^ The activities of mutual aid groups in the context of the current COVID‐19 crises might therefore require further consideration. Moving beyond the dominant paradigm which presents the narrow view of VI implies also appreciating and illustrating the potential of VI to reciprocally expand where we see the boundaries of PPI, and to be seen as part of a civil society construct. Another similarity, confirming the close link between VI and PPI, is the articulated role for both in shifting the power balance between lay people and professionals in any organisation where either may be found. This can be seen for VI when volunteers are acknowledged as being involved alongside public and statutory services^50^ and for PPI, seen in identifying them as contributing to ensuring professionals act in ways that will benefit the public.

The similarities of PPI and VI discourses identified here suggest that many more forms of VI can be conceptualised as integrated with PPI if PPI is conceptualised as VI, within a ‘civil society paradigm’. This paradigm has its academic roots in political science and sociology,^46^ ^(p. 178)^ helping resolve many of the challenges to our understanding their observed nature as being related to the drive to democratise the NHS, with PPI centrally needing to be seen as driven by volunteers and not other stakeholder groups, if this drive is to be presented as authentically realised.

### 4.3 Moving policy forward to meet the practice and theory demands of both activities

By comparing policy discourses which frequently situate PPI activity as distinct from VI with experiential accounts of VI we are able to highlight the discursive links, and disconnects, between the language of policy and the pragmatic activity of VI. Gee suggests that language can be used to ‘enact activities, perspectives, and identities’.^4^ ^(p. 4)^ Here there is a disconnect between policy and practice and we argue that there is the potential to position PPI as VI and more forms of VI as PPI. It then becomes possible to interrogate more rigorously the theoretical debates which frame both activities. However, such positioning also has direct implications for practice, as seen in the frequently debated tensions between professional and public roles and safety for all.^7^

A common trope in PPI and VI discourse is the reiterated tension around whether the public voice is respected and acted on by professionals and whether professionals may feel threatened by lay people activities infringing on their professional roles and judgements. PPI accounts commonly affirm tokenism on the part of professional service providers enacting the letter of the policy of public involvement but ignoring its lived meaning.^10^ Parallel concerns are also commonly expressed that volunteering activity may inappropriately lead to de‐professionalising paid staff while professionalising volunteers, replacing formal job roles and raising various safeguarding issues for volunteers, patients and their families.^50^ Within Case Study 2, we saw the example of the volunteer presenting themselves as at first working unquestioningly under the ‘expert’s’ direction. In Case Study 1, the volunteer was recognised as the expert in their contribution and the account conveys more of a sense of partnership in practice between professionals and the volunteer. Volunteer managers in health and social care settings have substantial experience and can access guidance on how to alleviate the tensions around recognising expertise for VI in health and social care. While such guidance appears applicable to PPI, it does not seem to be applied while PPI and VI are managed and valued as distinct.

In the PPI‐related discourses, while encouraging good practice to better achieve the benefits of public involvement, the requirement to safeguard people carrying out activities does not seem as prevalent as in the VI‐related discourses. Safety for the public is well‐defined and prioritised in volunteering^51^, ^52^ but there appears to be scope to consider safety in more depth in PPI. Indeed safety is a two‐way process, a well‐established concept in the VI discourse, because where the public are in contact with patients there is a wider organisational responsibility to protect patients and staff.^53^ Lessons learned from investigations into matters arising from the prominent recent examples of reported volunteering‐related sexually abusive activities, conclude that repeating such abuses can only be avoided if all volunteer involving organisations can refer to and act on a clear shared understanding. ‘It requires repeated reinforcement of messages, awareness ‐ raising and training, as well as regular ongoing testing of the effectiveness and relevance of safeguarding arrangements’.^53^ ^(pp. 121‐122)^

Despite these stark warnings afforded by the VI discourse, safety was rarely a feature of the PPI discourse reviewed. If we consider PPI as a type of VI, this immediately opens connections to an existing extensive body of practice guidance to guide such practical improvements in PPI providing more robust and tested means for transparently and accountably responding to such problems, putting pre‐emptive measures in place to mitigate their risks.

## 5 CONCLUSION

This analysis confirmed discursive disconnections between the current framing of PPI and of VI, both within and also across key health and social care organisations. This was seen to frame further disconnections between PPI and VI so obscuring their shared purposes and limiting their translation into practice. These disconnects may therefore help explain much of the common confusion in their related terminology. They can also be seen as creating knowledge disjunctures in deploying these distinct discourses, shown here to undermine the quality, availability and applicability of practice. These lead to common criticisms of both VI and PPI policy and practice which are argued here as justifiable.

PPI and VI are complex activities through which to extend collaborations with people from groups not ordinarily part of service and researcher organisations. Articulating either PPI or VI as separate will engender persistent uncertainties in meanings which if aligned can make them available to both. Such alignment may incidentally enable discussing and negotiating agreements on the specific nature of these activities. Applying a critical discursive analytical approach to policy and personal accounts is seen here to help refocus some uncertainties, so as to foreground the similarities and alliances between both types of activities, within health and social care settings. We illustrated how this helped reframing of the policy and practice focus to present PPI members as volunteers and to present volunteers as much more than simply ‘alternative deliverers’ of services but having active roles in reshaping them. To ensure more explicit sharing of meanings between both policy contexts, we need to reposition textual discourses from the routinely repeated statements of policy intent towards policy statements which are more securely grounded within the evidence that can identify PPI and volunteering as having similar practice concerns. Such similar concerns can then be understood to similarly motivate and so to realise similar democratic purposes.

Repairing the disjunctures between the two discourses of PPI and VI, by recognising the conceptual alignment of PPI as a form of VI, can powerfully resolve many contemporary widespread conceptual uncertainties. Doing this would also immediately represent and make available a body of robust good practice in VI to link to PPI purposes and practices. The corollary is that extending the VI discourse to incorporate a civic society paradigm, which already includes PPI, helps strengthen the rationale for more PPI, now seen as a policy imperative. Reconnecting these discourses as argued here can therefore more securely embed PPI and VI within the NHS constitution as our explicit right, affirming a health and social system which not only belongs to us all but which involves us all.

## DISCLAIMER

The views expressed are those of the authors, and not necessarily those of the NIHR, NHS or Department of Health and Social Care.

## PATIENT AND PUBLIC CONTRIBUTION

The idea for this review was developed through discussion with lay advisory members of the [Institute for Volunteering Research] during informal review of National Health Service [NHS] policies and workshops undertaken to explore the place of VI in health and social care. Two of the people providing accounts in this paper had helped to conceive the central proposition of the paper and also collaborated as co‐authors, reviewing manuscripts and providing critical commentary as the paper progressed and on this resubmission.

## CONFLICTS OF INTEREST

The authors have no conflict of interest to declare.

## AUTHORS’ CONTRIBUTION

JG conceived the presented idea, and carried out and reported the policy document collection. All five authors were involved in the initial design and overall study management. JG, LB and FP were involved in all stages of analysis planning and interpretation. All authors were involved in the drafting, editing and/or reviewing of this manuscript. HE and ML provided case accounts in this paper, helped to conceive the central proposition of the paper, contributing to all author activities except the initial stages of analysis of case accounts, reviewing manuscript versions and providing critical commentary as the paper progressed and on this resubmission.

## Data Availability

The materials that support the findings of this study are publicly available. We have included citations in the reference section.
